# Large Scale Profiling of Protein Isoforms Using Label-Free Quantitative Proteomics Revealed the Regulation of Nonsense-Mediated Decay in Moso Bamboo (*Phyllostachys edulis*)

**DOI:** 10.3390/cells8070744

**Published:** 2019-07-19

**Authors:** Xiaolan Yu, Yongsheng Wang, Markus V. Kohnen, Mingxin Piao, Min Tu, Yubang Gao, Chentao Lin, Zecheng Zuo, Lianfeng Gu

**Affiliations:** 1 Basic Forestry and Proteomics Research Center, College of Life Science, Fujian Provincial Key Laboratory of Haixia Applied Plant Systems Biology, Fujian Agriculture and Forestry University, Fuzhou 350002, China; 2 Jilin Province Engineering Laboratory of Plant Genetic Improvement, College of Plant Science, Jilin University, 5333 Xi’an Road, Changchun 130062, China; 3 Department of Molecular, Cell & Developmental Biology, University of California, Los Angeles, CA 90095, USA; 4 Basic Forestry and Proteomics Research Center, College of forestry, Fujian Agriculture and Forestry University, Fuzhou 350002, China

**Keywords:** *Phyllostachys edulis*, label-free quantitative proteomics, long noncoding RNA, alternative splicing, protein isoform, nonsense-mediated mRNA decay

## Abstract

Moso bamboo is an important forest species with a variety of ecological, economic, and cultural values. However, the gene annotation information of moso bamboo is only based on the transcriptome sequencing, lacking the evidence of proteome. The lignification and fiber in moso bamboo leads to a difficulty in the extraction of protein using conventional methods, which seriously hinders research on the proteomics of moso bamboo. The purpose of this study is to establish efficient methods for extracting the total proteins from moso bamboo for following mass spectrometry-based quantitative proteome identification. Here, we have successfully established a set of efficient methods for extracting total proteins of moso bamboo followed by mass spectrometry-based label-free quantitative proteome identification, which further improved the protein annotation of moso bamboo genes. In this study, 10,376 predicted coding genes were confirmed by quantitative proteomics, accounting for 35.8% of all annotated protein-coding genes. Proteome analysis also revealed the protein-coding potential of 1015 predicted long noncoding RNA (lncRNA), accounting for 51.03% of annotated lncRNAs. Thus, mass spectrometry-based proteomics provides a reliable method for gene annotation. Especially, quantitative proteomics revealed the translation patterns of proteins in moso bamboo. In addition, the 3284 transcript isoforms from 2663 genes identified by Pacific BioSciences (PacBio) single-molecule real-time long-read isoform sequencing (Iso-Seq) was confirmed on the protein level by mass spectrometry. Furthermore, domain analysis of mass spectrometry-identified proteins encoded in the same genomic locus revealed variations in domain composition pointing towards a functional diversification of protein isoform. Finally, we found that part transcripts targeted by nonsense-mediated mRNA decay (NMD) could also be translated into proteins. In summary, proteomic analysis in this study improves the proteomics-assisted genome annotation of moso bamboo and is valuable to the large-scale research of functional genomics in moso bamboo. In summary, this study provided a theoretical basis and technical support for directional gene function analysis at the proteomics level in moso bamboo.

## 1. Introduction

Bamboo belongs to gramineous plants with about 90 genera and 1200 species [1]. It is a major non-wood forest species and widely distributed in tropical, subtropical and temperate zones [2]. Moso bamboo has been cultivated for a long time and is one of the main bamboo species in China, and is rich in lignocellulose [3]. Its planting area accounts for 70% of Chinese bamboo and 80% of total bamboo timber [1]. Moso bamboo characteristically experiences rapid growth [1,2,3,4]. In addition, moso bamboo has many important economic, cultural and ecological values, such as edible bamboo shoot, timber, ornamental, and greening values [5,6,7]. The bamboo forest has an important ecological value for the terrestrial carbon cycle and for ecosystem protection [5,6,7]. Because of its important economic value and ecological perspectives, it has attracted the attention of community forestry, and has been regarded as the main model plant in the study of plant biology of bambusoideae.

The completed genome sequences of moso bamboo in 2013 have shown that 2.05 Gb assembled sequences included annotated 31,987 genes [4]. The annotated genes of the moso bamboo were based on prediction algorithms, and were further improved by the long-read sequencing technology of Pacific Biosciences (PacBio) [8,9]. In recent years, large-scale transcriptome data have also been available for moso bamboo [9,10,11,12,13,14,15,16], which greatly improved the annotation of protein-coding genes in moso bamboo. In additional to protein-coding genes, long noncoding RNA (lncRNA), plays an important regulatory role in the biochemical processes of organisms, although the function of this has not yet been identified in organisms [17,18]. In total, 1989 long non-coding RNAs (lncRNA) were predicted in moso bamboo [9]. Although the transcriptome greatly promotes the annotation of the moso bamboo genes, these hypothetical genes are based on gene prediction algorithms. Gene prediction algorithms are based on universal translation rules. The genome annotation process usually includes many errors and is generally inaccurate [19,20,21,22,23]. Recently, proteomic data from mass spectrometry experiments have been commonly used to correct and validate protein coding genes and identify new coding genes [21,22,23,24,25,26]. Proteomics also can effectively quantify the most valuable protein participants in the organism [21,22,23,27]. However, the high content of lignification and fiber in moso bamboo has caused difficulties in protein extraction, which limits the research progress of quantitative proteomics in moso bamboo [4,28,29]. Thus, the annotated gene according to the genome and transcriptome has not been validated from the protein level, and the function of these genes in the growth and development of moso bamboo remains unknown.

In eukaryotes, a single gene can produce multiple transcriptional isoforms through variable post-transcription regulation [30,31,32]. Next-generation sequencing (NGS) methods revealed multiple splicing isoforms, which were generated from alternative splicing (AS) [30,31]. In recent years, the PacBio sequencing platform, a single-molecule sequencing technology, can produce longer reads, greatly helping to recognize multiple splicing isoforms produced by AS in plant [33]. Full-length splice isoform sequencings have been applied to the discovery of multiple transcriptional isoforms produced by AS in a variety of species [15,34,35,36]. Similarly, previous studies have shown that AS and alternative polyadenylation (APA) were ubiquitous in moso bamboo [8,9,37]. The functional characteristics of protein isoforms translated by different splicing isoforms in eukaryotic organisms have been shown to be significantly diverse [38]. However, it remained unknown that if these splicing isoforms in moso bamboo could be translated into different protein isoforms because alternative transcripts might be degraded by nonsense-mediated mRNA decay (NMD) in mammalian [39] and plant [40,41,42]. In vitro expression experiments provide evidence for whether different splicing isoforms produced by the same gene encode proteins, and protein-protein experiments demonstrate a significant divergence in function between different isoforms [43]. Correspondingly, proteomics is also commonly used to identify whether different splicing isoforms encode proteins [44,45,46,47,48,49,50,51,52]. Compared to in vitro expression assays, evidence from the proteomics can more directly and strongly demonstrate whether different isoforms naturally encode proteins in vivo. However, it is unclear whether all isoforms in moso bamboo are identified by PacBio sequencing encode proteins, since there is a lack of evidence of protein levels. Proteome experiments are urgently needed to prove whether isoforms encode proteins.

Moso bamboo is an important forest species with a variety of ecological, economic and cultural values. However, the gene annotation information of moso bamboo is based on the transcriptome sequencing, lacking the evidence of proteome. In this study, we successfully optimized the extraction efficiency of moso bamboo protein and identified the total protein of moso bamboo by using label-free quantitative proteomics. These studies improved the annotation of the protein isoforms of moso bamboo based on proteomic analysis and revealed the differences in physiological process of moso bamboo by analyzing the protein expression profile of moso bamboo seedlings and leaves using label-free quantitative proteomics. Also, we proposed that 18% unique transcript isoforms included premature termination codons could also be translated into proteins. In summary, proteomic analysis in this study improves the proteomics-assisted genome annotation of moso bamboo and is valuable for the large-scale research of functional genomics in moso bamboo.

## 2. Materials and Methods

### 2.1. Plant Materials and Growth Conditions

**Figure 1:**
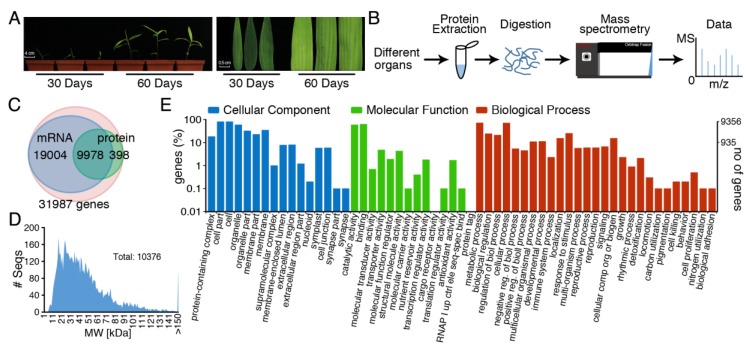
The flowchart of high-throughput label-free quantitative proteomics in moso bamboo. (**A**) The fully developed leaves of 30-days-old seedlings and 60-days-old moso bamboo were collected to detect the concentration and efficiency of protein extraction among different periods; (**B**) The pipeline of label-free quantitative proteomics; (**C**) Venn diagram of moso bamboo proteins and the mRNA transcription; (**D**) The distribution of molecular weight of moso bamboo proteins; (**E**) GO functional annotation of total proteins.

Moso bamboo (*Phyllostachys edulis*) seeds were treated with a shelling machine and soaked in water for 24 h. Seeds were sown on soil and grown in the greenhouse at a temperature of 25 °C and 16 h light/8 h dark photoperiod, with a light intensity of 80 μmol m^−2^ s^−1^ and 50% relative humidity. All the materials were in the same condition and were placed in the greenhouse for cultivation. The leaves, roots and stems of the moso bamboo were collected from 30- and 60-days period, respectively. All collected samples were rapidly frozen in liquid nitrogen and then stored at −80 °C. We collected a group of leaves with same sizes from multiple planting pots and pooled them together. The leaves collected during the 30-days and 60-days period were fully developed leaves (Figure 1A). In the process of material selection, we randomly selected each seedling. Multiple leaves with the same height and size were pooled together, which could ensure the reliability of the experimental results and enough material for protein extraction.

### 2.2. Extraction of Proteins from Moso Bamboo

Moso bamboo materials were frozen in liquid nitrogen and were extracted from ground material with lysis buffer (10 mM Tris-HCl pH 8.0 5 mM EDTA 1% SDS 8 M Urea 20 mM Dithiothreitol). The lysis buffer was supplemented with EDTA-free Protease Inhibitor Cocktail Tablets. After incubation on ice for 30 min, the lysate was centrifuged at 12,000× *g* for 15 min. After adding phenol extraction buffer, the supernatants were precipitated overnight at −20 °C using six volumes of 10% TCA/acetone (10% trichloroacetic acid dissolved in acetone) or 100% acetonitrile (ACN). After centrifugation at 12,000× *g* for 30 min at 4 °C, pellets were washed three times with pre-cooled acetone and dried for 5 min by vacuum freeze-drying. Protein samples were resuspended in buffer (8 M urea 0.1 M Tris-HCl pH 8.0). The concentration of purified proteins was measured by BCA protein assay (Thermo Fisher Scientific, Waltham, MA, USA) at the absorbance 562 nm, using bovine serum albumin as the standard.

### 2.3. Protein Digestion and Peptide Fraction

A total of 500 μg proteins from seedlings and leaves were digested using a filter-aided sample preparation (FASP) method following DTT reduction, iodoacetamide (IAA) alkylation and overnight digestion with trypsin at a ratio of 1:100. Digested peptides were dried using a CentriVap (Thermo Fisher) and pre-fractionated with Ultimate 3000 (Thermo Fisher scientific, Waltham, MA, USA). The peptide mixture was re-dissolved in buffer A (buffer A: 20 mM ammonium formate in water, pH 10.0, adjusted with ammonium hydroxide), and then fractionated by high pH separation using Ultimate 3000 system connected to a reverse phase column (XBridge C18 column, 4.6 mm × 250 mm, 5 μm (Waters Corporation, Milford, MA, USA)). High pH separation was performed using a linear gradient with 5% buffer B up to 45% buffer B over 40 min (buffer B: 20 mM ammonium formate in 80% ACN, pH 10.0, adjusted with ammonium hydroxide). The column was re-equilibrated to initial conditions for 15 min. The column flow rate was maintained at 0.8 mL/min and column temperature was maintained at 30 °C. In total, twelve fractions were collected. Each fraction was dried and desalted using a C18 Ziptip (Thermo fisher scientific).

### 2.4. Mass Spectrometry Analysis

Peptide fractions were resuspended with 30 μL solvent C (C: water with 0.1% formic acid) and analyzed by on-line nanospray LC-MS/MS on an Orbitrap Fusion coupled to an EASY-nano-LC system (Thermo Scientific, MA, USA). For one LC-MS/MS run, 5 μL (1 μg) peptide sample was loaded onto the trap column (Thermo Scientific Acclaim PepMap C18, 100 μm × 2 cm), with a flow of 10 μL/min and subsequently separated on the analytical column (Acclaim PepMap C18, 75 μm × 15 cm) with a 60-min linear gradient of 3–32% solvent D (D: ACN with 0.1% formic acid). The column flow rate was maintained at 300 nL/min. The electrospray voltage of 2 kV versus the inlet of the mass spectrometer was used.

The mass spectrometer was run under data dependent acquisition mode, and automatically switched under MS and MS/MS mode in 3 s cycles. MS1 mass resolution was set as 60 K with *m*/*z* 350–1550 and MS/MS resolution was set as 50 K under HCD mode. The dynamic exclusion was set to n = 1, and the dynamic exclusion time was 45 s. AGC target was 8 × 10^4^ and the max injection time was 120 ms.

### 2.5. Protein Identification Using Mass Spectrometry

Open reading frames (ORFs) were extracted and translated into amino acid sequences using TransDecoder software (v.5.5.0) with the default option [53]. This software is also used to build the potential amino acid sequences for 1989 long chain non-coding sequence and 93,481 unique transcript isoforms [9]. All MS/MS raw data were analyzed using Proteome Discoverer 2.1 (Thermo Fisher Scientific, San Jose, CA, USA; version 2.1) with the SEQUEST algorithm. A total of 31,987 protein-coding genes have been annotated in the moso bamboo genome [4]. In this study, we used annotated protein sequence from previous published paper [4], which is a combination of genome and transcriptome sequencing analysis of the encoded information of the moso bamboo. That protein database provided one most reliable and unique protein annotation for each locus. All MS/MS spectra searches were conducted against a concatenated forward/reversed version of *P. heterocycla-v1.0* sequence database [4] and filtered using a false discovery rate (FDR) < 0.01 at both the peptide and protein level. Carbamidomethyl of cysteine were selected as the fixed modification while oxidation (M), acetylation (protein N-term) and phosphorylation (STY) were allowed for variable modifications. The database search was performed with an initial precursor ion tolerance of 10 p.p.m. Fragment ion mass tolerance to 0.02 Da and two missed cleavages are allowed. Target decoy searches strategy provided a substantial improvement in large-scale protein identification by mass spectrometry [54]. For label-free procedures, protein abundance quantification was carried out based on the identified peptides using the scaffold software [55]. The relative expression abundance of proteins were calculated and normalized as spectral abundance factors (NSAF) label-free quantitative algorithm which based on the spectral counts (SpC) to define the relative expression of the proteins as SpC divided by the protein length (L), and divided by the total SpC/L across all identified proteins [56,57,58]. The protein quantitation and confidence were performed using a relatively conservative threshold (fold change ≥ 4 for up-regulation or ≤ 0.25 for down-regulation), false discovery rate < 0.01 with at least 2 peptides matched were considered for further analysis).

### 2.6. GO Enrichment Analysis and Pathway Analysis

Blast2GO software optimizes the BLAST algorithm to identify homologous sequences and then assigns uncharacterized novel sequences in non-model species with functional annotations, which is represented via the gene ontology (GO) vocabulary [59]. In this study, GO terms of each gene in moso bamboo were assigned using BLAST2GO with default option, and GO terms was assessed with InterProScan [59]. The enrichment of GO terms was assessed with the BiNGO plugin for Cytoscape, using hypergeometric test for statistical analysis. The cutoff *p value* is set to 0.05 and all terms with a *p* value < 0.05 were considered statistically as over-represented GO and shown as colored nodes in the enrichment network and histogram. Pathway analysis were performed using KEGG online sites (https://www.kegg.jp/blastkoala/) [60].

### 2.7. Data Availability

The original LC-MS/MS file was available at http://forestry.fafu.edu.cn/pub/cells and could be analyzed by protein retrieval software such as Proteome Discoverer.

## 3. Results

### 3.1. Extraction and Identification of Total Protein Using Phenol Extraction Combined with Acetonitrile Precipitation for Orbitrap Fusion Tribrid Mass Spectrometry

In this study, proteins of 30-days-old seedlings and 60-days-old leaves were extracted, and their concentration was analyzed with the BCA assay. The fully developed leaves of 30-days-old seedlings and 60-days-old moso bamboo were collected to detect the concentration and efficiency of protein extraction among different periods (Figure 1A). Phenol extraction combined with acetonitrile precipitation or TCA/acetone precipitation were used to extract bamboo protein. For stem and root sample harvested at 60-days old, phenol extraction combined with acetonitrile precipitation resulted in only low protein concentrations due to serious lignification and increased fiber content at this developmental stage. Nevertheless, this method could effectively extract total proteins from 30-days-old seedlings and 60-days-old leaves (Figure 1A). We did not carry out mass spectrometry analysis for the roots and stems of 60-days-old moso bamboo because of the low protein concentration. Protein samples extracted from seedlings and leaves of moso bamboo were analyzed using Orbitrap Fusion Tribrid mass spectrometry (Figure 1B). In total, 1,020,880 tandem mass spectra were generated (Table 1).

Comparing the observed spectra with the protein database derived from prediction of genome and transcriptome data, 55,200 peptides were identified from these highly trusted mass spectrometry data. In total, mass spectrometry peptides matching identified 10,376 annotated genes, which further verified the gene models on a translational level in moso bamboo (Figure 1C). The protein molecular weight map showed that the 10,376 detected proteins ranged from 10 kDa to 150 kDa. The protein content was relatively high in the range of 15 kDa to 90 kDa, which covered a wide protein size distribution (Figure 1D). Phenol extraction combined with acetonitrile precipitation method could extract 7192 and 9336 proteins from seedlings and leaves of moso bamboo, respectively. However, phenol extraction combined with TCA/acetone precipitation only extracted 1937 and 1635 proteins from seedlings and leaves, respectively. The results further showed that phenol extraction combined with acetonitrile precipitation method was efficient and feasible for extracting total protein from moso bamboo (Table 1).

### 3.2. Analysis of Physiological Process and Metabolic Pathway for All Identified Protein Data

GO functional annotation and classification were performed for all identified protein data of moso bamboo by using Blast2GO software [59]. Metabolic process, cellular process and response to stimulus occupied the first three positions in the biological process, indicating that the proteins involved in these physiological metabolic processes were relatively high in the expression of protein (Figure 1E). GO terms from the molecular function ontology shown that most genes were involved in binding, catalytic activity, transporter activity and structural molecule activity. The results of GO annotation analysis showed a general physiological state of moso bamboo.

### 3.3. Differential Expression Profiles of Proteomics in Seedlings and Leaves of Moso Bamboo using High-Throughput Label-Free Quantitative Proteomics

**Figure 2:**
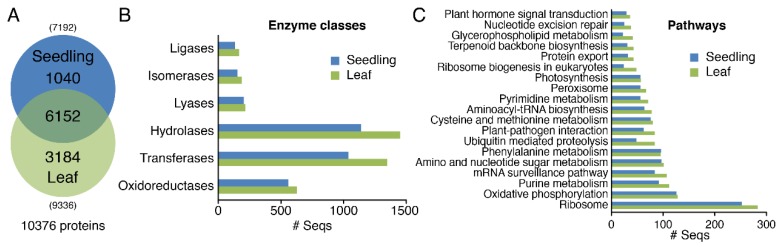
Profiles of proteins in seedlings and leaves of moso bamboo. (**A**) Comparison of protein numbers in seedlings and leaves of moso bamboo; (**B**) The classification of enzymes in seedlings and leaves of moso bamboo; (**C**) Pathway analysis, according to the Kyoto Encyclopedia of Genes and Genomes (KEGG) database.

A total of 7192 and 9336 proteins were identified in 30-days-old seedlings and 60-days-old leaves, respectively. 1040 proteins were specific to the seedling stage and 3184 proteins were specifically identified in the 60-days-old leaves (Figure 2A). In addition, 6152 proteins were identified in both tissues, accounting for almost 60% of all identified proteins, 85% of the identified proteins in 30-days-old seedlings and 66% of the identified proteins in 60-days-old leaves. The results showed that the physiological and biochemical processes were similar in the leaves and seedlings of moso bamboo.

According to all the identified proteomic data of moso bamboo, the functional enzymes were classified and the data on the seedlings and leaves of moso bamboo were compared. The common enzyme classification mainly included oxidoreductase, transferase, hydrolase, lyase, isomerase and ligase. The three most frequent enzyme classes were transferase, hydrolase and oxidoreductase, which indicated that the biological processes of moso bamboo mainly included oxidation-reduction processes, amino acid metabolism processes and tricarboxylic acid cycle reactions. Interestingly, the number of enzyme classification for leaves was higher than that for seedlings. It may be that the physiological metabolism process in the leaves of moso bamboo is more active than the seedlings (Figure 2B).

The analysis of the metabolic pathways indicated that the proteins identified in the seedlings and leaves mainly included ribosome, oxidative phosphorylation and purine metabolism. Although the identified proteins in the leaves and seedlings of moso bamboo were part of the same metabolic pathways, the number of proteins in the same metabolic pathway was different. In all metabolic pathways, the number of proteins enriched in the leaves of moso bamboo was higher than that of the seedlings (Figure 2C). Other important pathways included photosynthesis and phenylalanine metabolic pathway. Oxidative phosphorylation was a kind of energy conversion of proteins, which occurred in mitochondria. The above results showed that part physiological metabolism was similar in the seedlings and leaves of moso bamboo, and there was also different physiological state. Comparing with seedling, physiological metabolism of the leaves was slow. However, the leaves activated and expressed more proteins related to plant stress response, disease defense and energy metabolism in order to ensure the normal physiological function and stress resistance of the organism.

### 3.4. GO Analysis of Proteomics in Seedlings and Leaves of Moso Bamboo

**Figure 3:**
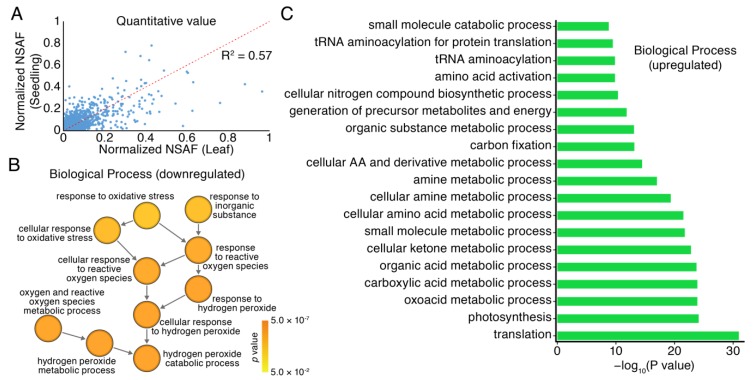
The gene ontology (GO) enrichment analysis of differential expression proteins in seedlings and leaves of moso bamboo based on mass spectrometry-based label-free quantitative proteomics. (**A**) The correlation analysis of protein abundance between seedlings and leaves of moso bamboo; (**B**) The biological process for down-regulated proteins; (**C**) The biological process for up-regulated proteins.

The correlation analysis of normalized spectral abundance factors (NASF) between 30-days-old seedlings and 60-days-old leaves had a correlation coefficient of 0.57 (Figure 3A), suggesting a moderate correlation between both tissues. These results indicated that moso bamboo had common as well as distinct physiological and biochemical processes at different growth stages. Biological processes of protein enrichment in seedlings and leaves of moso bamboo were similar with each other. There was no significant difference in the number of proteins involved in several biological processes, including glycolysis, translation and membrane transport. Moso bamboo presented as being fast-growing, and the fully developed leaves from 30-days and 60-days was significantly different in the size and color (Figure 1A). Thus, the correlation coefficient between 30-days-old seedlings and 60-days-old leaves was 0.57, suggesting a variation between two periods. Compared with the leaves from 30-days-old seedlings, there were 1011 differentially expressed proteins in the 60-days-old leaves, accounting for 22.1% of the total number of proteins quantified. Among 1011 differentially expressed proteins, 37 were down-regulation and 974 were up-regulation in 30-days-old seedlings compared to that in 60-days-old leaves.

In addition, we analyzed the differences in protein expression profiles and performed GO enrichment analysis on the differentially expressed proteins by using label-free quantitative proteomics with a *p* value cutoff < 0.05 in moso bamboo from 30-days-old and 60-days-old fully developed leaves, respectively (Figure 3B,C). Enrichment biological processes in down-regulated proteins in seedlings compared to leaves were mainly involved in GO terms related to hydrogen peroxide catabolism. Regulation of hydrogen peroxide was often associated with plant stress responses and disease defenses. Specific binding of kinase and substrate play a role in regulating plant growth and development, and are also key regulators of signal transduction [61]. The number of proteins involved in redox process and oxidative phosphorylation in leaves was more than that in seedlings, which indicated that more energy might be needed to maintain the balance of the organism in the later growth stage of moso bamboo (Figure 3B). Enrichment biological processes in up-regulated proteins included amongst others translation and photosynthesis (Figure 3C).

### 3.5. Discovery of New Protein Coding Genes

**Figure 4:**
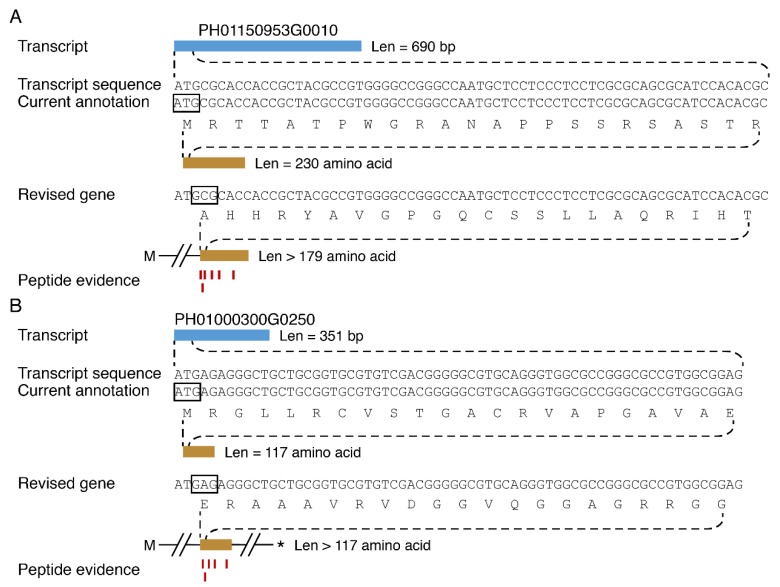
Revision of gene annotation in moso bamboo and discovery of new genes. Current annotation and translation frame (black frames) and revised gene models of PH01150953G0010 (**A**) and PH0100300G0250 (**B**) supported by matching peptide spectra. Stop codon was marked by an asterisk.

A comparison of all protein database produced by the mass spectrometer with the current annotated protein database created from genomic and transcriptome data revealed that many tandem mass spectra were not matched, which reminded us that the genome annotation information of moso bamboo is not completely accurate. To modify and supplement the coding information of moso bamboo genome, the remaining 21,611 unproven-gene annotation genes were translated using all potential open reading frame and were matched with tandem mass spectra to identify new genes and annotated coding genes. According to internationally recognized standards for protein mass spectrometry identification, each recognized protein had at least two or more peptides matching support. In total, we identified 1108 new translation frames, and this translation information complemented the previously predicted gene coding patterns (Figure 4).

**Figure 5:**
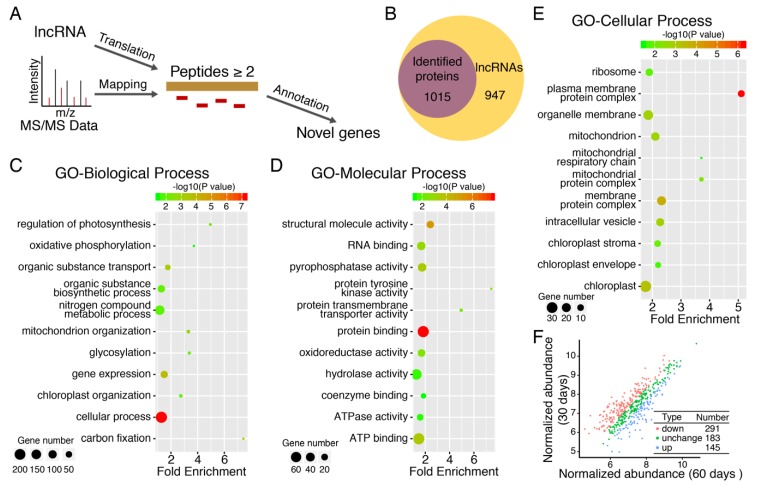
The re-annotation of lncRNA through mass spectrometry data. (**A**) Overview of pipeline for lncRNA re-annotation. All lncRNAs were translated into amino acid sequences. Selected Amino acid (aa) sequences (> 100 aa) were compared to mass spectrometry data and sequences with. more than 2 peptide-matches were considered to be authentic; (**B**) Venn diagram illustrating the number of lncRNA re-annotated by mass spectrometry; (**C**) The biological process for lncRNA re-annotated by mass spectrometry; (**D**) The molecular function for lncRNA re-annotated by mass spectrometry; (**E**) The cellular component for lncRNA re-annotated by mass spectrometry. Explanatory information on the functional enrichments and numbers of involved proteins in terms were all listed on the left. The cutoff of the *p* value is set at 0.05. Different colors (such as red, green and yellow) stand for –log10 (*p* value) in the GO analysis; (**F**) The plot of differentially expressed lncRNA encoded proteins.

In addition, 1989 locus were predicted to be non-coding RNA in the transcriptome analysis of moso bamboo using PacBio long reads sequencing [9]. Since these lncRNAs were identified based on software prediction, parts of the RNA sequences might actually encode proteins. In order to test this hypothesis, all lncRNAs were translated and matched against tandem mass spectra (Figure 5A). Among the 1989 predicted lncRNA, 1015 original proteins were successfully identified by mass spectrometry. This shows that translational activity could be validated for about half (51.03%) of the in-silico predicted lncRNA (Figure 5B). We also performed the differentially expressed lncRNA encoded proteins and identified 291 down-regulated and 145 up-regulated protein, respectively (Figure 5F). The newly identified 1015 proteins-encoding genes greatly complement the moso bamboo gene annotation database, which laid the foundation for future studies of moso bamboo. The re-annotation according to mass spectrometry data had obvious advantages over that provided by prediction, which could accurately predict the correct translation framework.

Homology and functional analysis of these new protein-coding genes were performed by BLASTP against NCBI’s non-redundant protein database (NR) using Blast2GO. InterProScan was used to annotate the functions of these new genes. Of the new genes coding translation frames, 971 genes were functionally annotated and classified and for 1211 genes, no annotation could be retrieved. Among the functional classification of annotated genes, 254 genes were involved in cellular processes, 164 genes were involved in nitrogen compound metabolic processes, and there were also many genes participated in carbon fixation, photosynthesis, response to stimulus and gene expression, etc. (Figure 5C–E).

### 3.6. Large Scale Identification of Protein Isoforms

**Figure 6:**
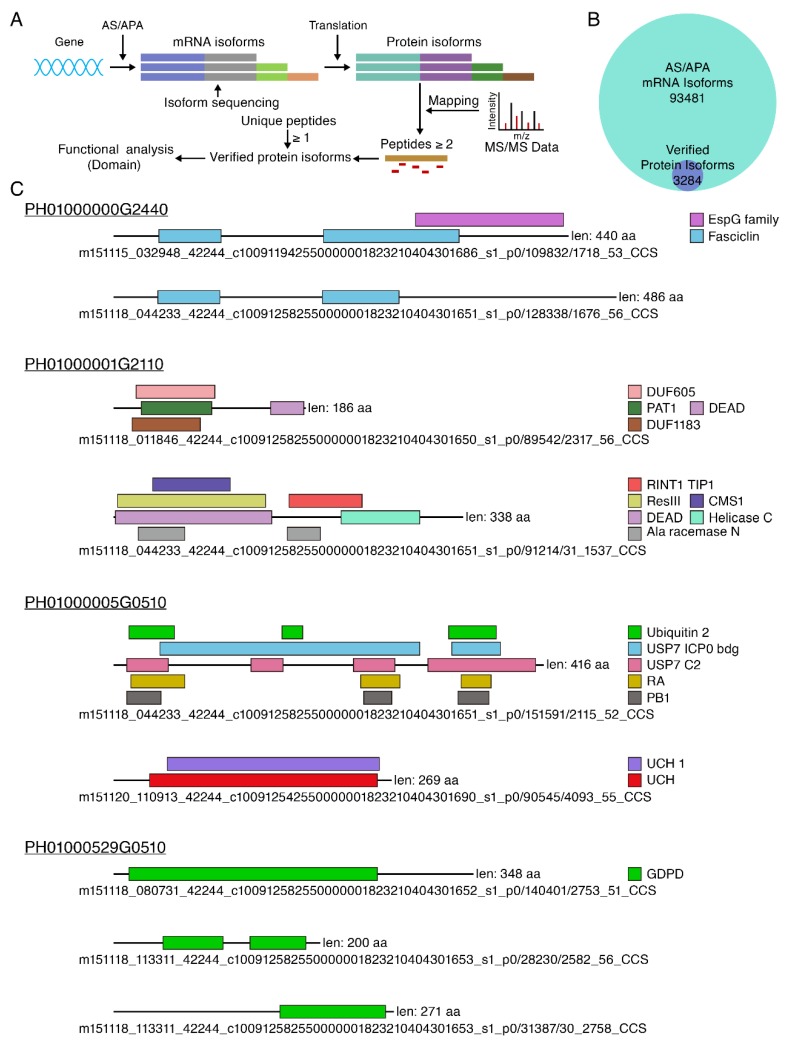
Different protein isoforms from the same locus were identified by mass spectrometry data. (**A**) The pipeline for the verification of different protein isoforms from the same locus for moso bamboo through protein profiling data; (**B**) The Venn diagram illustrating the number of different protein isoforms verified by mass spectrometry in all AS/APA isoform; (**C**) Domain analysis of different protein isoforms from the same locus.

We analyzed whether the 71,923 and 82,390 unique alternative splicing and alternative polyadenylation isoforms, respectively, from 12,091 genes obtained by PacBio Iso-sequencing encoded proteins, could be mapped with mass spectrometry data [9]. In total, 93,481 unique isoforms could be translated into 163,669 potential amino acid sequences by TransDecoder software (Figure 6A). Among all different transcript isoforms matched to mass spectrometry data, we verified that 3284 unique transcript isoforms from 2663 genes could encode proteins, accounting for 3.51% of all isoforms (Figure 6B). The low percentage might be caused by expression levels below the detection threshold for some of the isoforms. Mass spectrometry matching 3284 unique isoforms provided evidence of how each isoform was encoded. Domain analysis of 3284 protein isoforms exhibited a variety of domains (Figure 6C): (1) Long protein isoform contains all domains of the short protein isoform, a total of 298 genes, such as PH01000000G2440; (2) Different protein isoforms from the same gene contained both the same and specific domains, with a total of 187 genes, such as PH01000001G2110; (3) Different protein isoforms from the same gene contain completely different domains, a total of 134 genes, such as PH01000005G0510; (4) Different protein isoforms from the same gene contained the same domain, a total of 230 genes, such as PH01000529G0510. These results suggest that different protein isoforms from one locus have similar or distinct functions due to alternative splicing.

The majority of alternative splicing-derived transcripts contain premature termination codons (PTCs), which are degraded by NMD pathways [62,63]. NMD is typically triggered only by stop codons at least ~50 nt upstream of the last exon–exon junction [64]. Among 93,481 unique transcripts isoforms, 20,825 were found to be NMD-regulated, accounting for 22.2% of all transcript isoforms. Among the 3284 protein-encoding isoforms identified by mass spectrometry, 601 splicing isoforms received NMD regulation, which accounted for 18.3% of all protein-encoding isoforms and suggested that part transcripts targeted by NMD might escape from the mRNA surveillance mechanism.

## 4. Discussion

Moso bamboo has important ecological, economic and cultural values, and also has the characteristics of strong carbon sequestration, rapid growth and tall plant. The combination of moso bamboo genome data and transcriptional data contributes to annotating moso bamboo genome [4,9]. However, few studies have supported annotated data of these moso bamboo genes through proteomes to form a more reliable and better annotated reference database. Proteome analysis based on high-resolution mass spectrometry can accurately help to establish and modify genome coding gene annotation information of species, which has been reported in many model plants. In *Arabidopsis thaliana*, proteome analysis was successfully applied to correct and improve annotation information of the genome [65,66]. In monocotyledon maize, 165 new protein-coding genes were annotated and 741 gene-coding models were modified by proteomics [67]. Similarly, in rice, proteome data and transcriptome data have greatly improved the annotation of rice genome [68,69]. However, few studies have supported annotated data of these moso bamboo genes through proteomes to form a more reliable and better annotated reference database. In this study, the extraction method of moso bamboo protein was optimized, hence the differences of different extraction methods for the extraction of moso bamboo protein were compared. It was found that phenol extraction combined with acetonitrile precipitation method had the best extraction effect. The extraction of moso bamboo by phenol extraction combined with acetonitrile precipitation method, a total of 10,376 proteins in moso bamboo were successfully identified, and the number of proteins identified at one time accounted for more than 30% of the number of all annotated genes (Figure 1C). The number of proteins identified by phenol extraction combined with acetonitrile precipitation is 5 times higher than of that with TCA/acetone precipitation (Table 1). This result showed that the extraction of moso bamboo protein by phenol extraction combined with acetonitrile precipitation was highly feasible. There are many methods for protein extraction, among which protein precipitation with miscible organic solvents (usually acetonitrile, methanol, acetone and ACN precipitation method) is the most commonly used. Of these extraction methods, acetonitrile precipitation method is the most effective to precipitate proteins, in which the vast majority of proteins were precipitated [70]. In this study, we found that the acetonitrile method was more efficient than the TCA/acetone method. Since the polar of acetonitrile is higher than that of acetone, it is expected to compete more successfully for water bound to protein, leaving more charged functional group available on protein aggregates to attract free amino acids from solution [71]. Thus, acetonitrile has a higher compatibility with water than acetone. In this study, acetonitrile presented more dehydrated and made it more susceptible to precipitation. Another reason may be that acetonitrile can better dissolve fiber compared with acetone.

In this study, phenol extraction combined with acetonitrile precipitation method was more efficient than TCA/acetone method. The establishment of efficient method for extracting the total proteins from leaves, as well as the identification of proteins based on mass spectrometry provided an accurate and unbiased method for the identification of proteins and improving annotation information in moso bamboo. Importantly, effective annotation of moso bamboo genome will cause some more extensive impacts on subsequent genetic improvement, gene function analysis, germplasm identification and the protection of moso bamboo. In this study, we also extracted the root and stem proteins of moso bamboo after 60-days. However, the concentration and efficiency of protein extraction were low. Label-free quantitative proteomics could not be carried out. Thus, the method of extracting protein from root and stem still needs further optimization.

In this study, we used proteomic data to validate and modify the gene annotation information of moso bamboo. The study of genome annotation and proteome of moso bamboo is helpful to the genetic breeding of moso bamboo and other species, with important scientific significance. In this study, we identified 55,200 peptides in the genome, which matched 10,376 predictive protein coding genes and validated the translation of predictive gene model. By matching peptides with all potential open reading frames of the corresponding gene sequences, we further improved 1108 gene models. Proteomic data could help us identify whether predicted lncRNA sequences were real non-coding sequences. Among other species, researchers corrected some predicted non-coding RNAs that actually encoded proteins by proteomic data [21,25]. In this study, the mass spectrometry peptides revealed that 1015 predicted lncRNAs might encode proteins.

Proteomics is also widely used to identify different protein isoforms from the same gene in various species [44,45,48,49,50,51,52]. In this study, 3284 transcript isoforms were verified to encode proteins through proteomic mass spectrometry data, accounting for 3.51% of all protein isoforms. In previous studies, only a small amount of alternative splicing proteins was verified by the proteomic method [48,49,50,51,52]. This may be mainly due to the sensitivity of current protein profiles and protein extraction methods. However, not all alternative splicing isoforms play important roles in organisms and are capable of encoding amino acids [50]. The majority of alternative splicing-derived transcripts contain premature termination codons (PTCs), which are degraded by nonsense-mediated mRNA decay (NMD) pathways [47,62,63]. NMD is a post-transcriptional quality control mechanism that selectively degrades mRNA containing PTCs [64,72,73]. In this study, mass spectrometry supported the translation of 18.3% transcript isoforms targeted by NMD. It will be interesting to investigate how these transcript isoforms escaped from mRNA surveillance mechanism. Alternative splicing can generate splicing isoforms targeted by NMD, which is a transcript quality control mechanism to regulate RNA turnover. This study suggested that isoforms with NMD regulation could also give rise to protein produces, which has been validated by label-free proteomics. For future studies that quantitative proteomics performed will be important for NMD factor mutants or for splicing factor mutants to investigate the interplay between NMD machinery and alternative splicing.

In vitro protein functional analysis experiments have demonstrated that different protein isoforms have diverse functions in organism [38,43]. Different protein isoforms play different roles in the organism, and the functional divergences are obvious [38,43]. For example, in humans the BCL-2 gene encodes for two protein isoforms with opposite functions: Bcl-x inhibits cell apoptosis, and Bak promotes cell apoptosis [74]. In this study, by analyzing domains from different isoforms of gene, the results suggest that different isoforms from the same gene have the same and specific functions in moso bamboo. In addition, in some different isoforms, the domains they contain are almost completely diverse, and the results strongly suggest that they play a completely diverse role in the growth and development of moso bamboo.

## 5. Conclusions

In this study, proteomics was used to improve and correct the protein coding information, which was previously annotated by genomic and transcriptomic data of moso bamboo. Here, we provided experimental evidence for predicted gene models and identified new protein-coding models, which further improved the gene annotation. Proteomic data can help us identify functions in many new transcripts of genome and transcriptome research. In addition, analysis of proteome can identify and correct some gene models that have been omitted by gene prediction algorithms. Interestingly, 18.3% transcript isoforms targeted by NMD could escape from the mRNA surveillance mechanism. The combination of proteome, transcriptome and genome analysis to improve the assembly and annotation of genomes of any species will become a model for future genome sequencing research. The proteome analysis in this study provided evidence for improving the annotation information of predictive genes in moso bamboo. Importantly, the proteomic evidence based on mass spectrometry provided an accurate and unbiased method for the identification of proteins in moso bamboo. The effective annotation of moso bamboo genome will cause some more extensive impacts on subsequent genetic improvement, gene function analysis, germplasm identification, and the protection of moso bamboo.

## Figures and Tables

**Table 1:** The comparative analysis of the efficiency of the protein extraction methods in seedlings and leaves of moso bamboo.

Phenol Extraction				
	Acetonitrile		Tca/Acetone	
	Seedling	Leaf	Seedling	Leaf
Spectra	255470	295637	243561	226212
Peptide	19470	30881	3155	1694
Protein	7192	9336	1937	1365
